# Severe vision impairment is associated with cumulative social risk among adults in Puerto Rico: a population-based study

**DOI:** 10.3389/fpubh.2026.1907768

**Published:** 2026-09-16

**Authors:** Mariola A. Busquets, Ibrahim D. Ali, Andrea N. Ponce, Jeremy D. Keenan, Luma Al-Attar

**Affiliations:** 1 School of Medicine, University of Puerto Rico, San Juan, PR, United States; 2 Department of Epidemiology, Johns Hopkins Bloomberg School of Public Health, Baltimore, MD, United States; 3 Francis I. Proctor Foundation, University of California, San Francisco, San Francisco, CA, United States; 4 Department of Ophthalmology, University of California, San Francisco, San Francisco, CA, United States; 5 Department of Ophthalmology, University of Puerto Rico, San Juan, PR, United States; 6 Retina Center of Puerto Rico, Manatí, PR, United States; 7 Johns Hopkins Bloomberg School of Public Health, Baltimore, MD, United States

**Keywords:** blindness, health equity, health surveillance, population-based, Puerto Rico, social determinants of health, vision impairment

## Abstract

**Introduction:**

Social determinants of health (SDoH) are increasingly recognized as drivers of population health, but their association with severe vision impairment has not been examined in Puerto Rico, a U. S. territory with a particularly high reported prevalence of severe vision impairment.

**Methods:**

We conducted a population-based, cross-sectional analysis of 4,558 noninstitutionalized adults aged ≥18 years who participated in the 2023 Behavioral Risk Factor Surveillance System (BRFSS) in Puerto Rico. Responses were dichotomized into five SDoH exposure variables representing Healthy People 2030 domains: economic instability, low educational attainment, insufficient social support, limited health care access, and unstable built environment. These were summed to construct a cumulative Social Risk Index (range, 0 to ≥4). Modified Poisson regression with robust variance estimation was used to estimate adjusted prevalence ratios (aPR) and 95% confidence intervals (CI) for the association between each exposure variable and self-reported severe vision impairment, adjusted for age, sex, marital status, and insurance coverage. Analytic weights were incorporated to account for the complex survey design.

**Results:**

Among 4,558 respondents (mean age, 50 years; 53.3% female), 880 reported severe vision impairment (survey-weighted prevalence, 19.8%). After adjustment, severe vision impairment was more prevalent among individuals experiencing an unstable built environment (aPR, 2.02; 95% CI, 1.70–2.40), limited health care access (aPR, 1.95; 95% CI, 1.53–2.50), economic instability (aPR, 1.68; 95% CI, 1.40–2.01), insufficient social support (aPR, 1.33; 95% CI, 1.10–1.61), and low educational attainment (aPR, 1.30; 95% CI, 1.06–1.60). A graded increase in prevalence was observed with higher cumulative Social Risk Index scores, from aPR 1.28 (95% CI, 1.03–1.59) for one social risk factor to aPR 3.00 (95% CI, 2.13–4.23) for four or more factors.

**Discussion:**

Self-reported severe vision impairment in Puerto Rico was associated with multiple individual social determinants and increased monotonically with cumulative social risk. Identifying social risk factors associated with severe vision impairment may help direct eye health resources toward the populations in which it is most prevalent. Further research is needed to clarify the mechanisms linking social determinants to vision loss.

## 1 Introduction

The social determinants of health (SDoH) are increasingly recognized as critical factors that shape health outcomes. The SDoH are defined as the conditions in the environments where people are born, live, learn, work, play, worship, and age (1). Addressing the SDoH is one of the five overarching goals of Healthy People 2030, a national initiative that establishes the United States (U. S.) public health agenda each decade. To effectively tackle these determinants, Healthy People 2030 categorizes the SDoH into five domains: economic stability, education access and quality, health care access and quality, built environment, and social and community support (2).

The association between SDoH and vision impairment has been demonstrated in a variety of settings in the U. S., but not in Puerto Rico (3–7). Examining SDoH in this context is important for identifying social and structural barriers to seeking and receiving care, which may be associated with delayed diagnosis and intervention. Puerto Rico warrants special attention due to several distinguishing features: a Spanish-speaking, aging population with distinct cultural norms; high rates of poverty; lower rates of advanced education; a fragmented health care infrastructure; and a distinct health insurance landscape with low uninsured rates, high rates of public health insurance, and high Medicare Advantage penetration (8, 9).

In 2022, the Behavioral Risk Factor Surveillance System (BRFSS) introduced an optional SDoH module for U. S. states and territories, designed to capture self-reported social risks through standardized questions (10). Prior studies have examined associations between SDoH and severe vision impairment using BRFSS data but had to rely on proxy measures for SDoH (3, 4). The relationship between visual impairment and the SDoH in this new module has not been comprehensively evaluated. Using 2023 BRFSS data, the present study leverages both the core questionnaire and the finalized SDoH module to assess vision health in Puerto Rico. To our knowledge, this is the first study to characterize severe vision impairment in Puerto Rico using population-based data. Our objective was to determine if various SDoH were associated with self-reported vision impairment in Puerto Rico, and to assess whether individuals with a greater number of social risk factors had a higher prevalence of severe vision impairment. Since the BRFSS provides cross-sectional data, the associations reported here describe co-occurrence rather than causation. Social risk may precede vision loss, may follow from it, or both, and this analysis cannot distinguish among these possibilities.

## 2 Methods

### 2.1 Design/sample

The CDC conducts the BRFSS (RRID: SCR_012974), a population-based, cross-sectional survey in all U. S. states and territories. As the world’s largest ongoing telephone-based health survey, the BRFSS collects information on health-related behaviors, chronic conditions, and preventive services among noninstitutionalized adults aged 18 and older (11). BRFSS data is de-identified and publicly available (12). The BRFSS draws a stratified random sample of landline telephone numbers and a random sample of cellular telephone numbers within each participating state and territory using Random Digit Dialing (13). The number of interviews within each state will vary based on funding and the size of regions, such as health districts, within each state. For every state or territory, final analytic weights combine a design weight with iterative proportional fitting (raking) to known population totals for demographic characteristics including age, sex, race and ethnicity, education, marital status, home ownership, and telephone source, so that weighted estimates are intended to represent the noninstitutionalized adult population (13, 14). The survey methodology has been described in detail elsewhere (14).

The survey, available in both English and Spanish, consists of a core set of required questions along with optional state-specific modules. Of particular relevance to this study is the opt-in 10-question SDoH module, which was piloted in 2017 and implemented for use by all states/territories in 2022 (10). The module serves as a surveillance tool for assessing directly reported SDoH. Since its official rollout, Puerto Rico has participated in 2022 and 2023. The 2023 BRFSS was selected for our analysis (15). All respondents were included in the study sample (*n* = 4,558). The 2023 Puerto Rico BRFSS response rate was 56.6%, compared with the median 2023 BRFSS response rate of 44.6% across all U. S. states and territories (16).

### 2.2 Measures

Most variables of interest, except education level, were extracted from the Social Determinants and Health Equity (SDHE) module. The main outcome was severe vision impairment, measured using the question from the core module: “Are you blind or do you have serious difficulty seeing, even when wearing glasses?” All questions in the BRFSS undergo technical review, cognitive testing, and field testing before being placed on the questionnaire (13). This item is the U. S. Department of Health and Human Services standard disability question on vision. It is carried in the BRFSS core questionnaire, is worded identically to the vision question in the American Community Survey, and has been used as the vision outcome in prior population-based analyses (3–5, 17). It is also the only measure of visual function in the BRFSS core questionnaire (15). In a validation study comparing telephone survey responses with best-corrected visual acuity determined by chart review, this survey question wording had the highest accuracy of the self-reported measures tested for identifying blindness, defined as best-corrected acuity of 20/200 or worse (18). Median best-corrected acuity among respondents answering yes fell between 20/80 and 20/200, compared with better than 20/40 among those answering no (18). The BRFSS does not collect examination-based confirmation of vision status.

The SDHE module was comprehensively reviewed to select measures corresponding to the following social risk factors based on the Healthy People 2030 SDoH domains: economic instability, low educational attainment, limited health care access, unstable built environment, and insufficient social support (Table 1). Alfaro Hudak et al. examined the same social factors; however, their analysis predated the implementation of the SDHE module (3). We sought to preserve measures used in their analysis to the greatest extent possible. However, due to differences between the SDHE module and the main BRFSS questionnaire, we incorporated new relevant measures to more comprehensively capture each social risk factor. Sociodemographic characteristics (e.g., age, sex, household income, and employment status) and health-related measures (e.g., health insurance coverage, having a personal health care provider, and general health status) acquired from the core module were examined to describe the sample and as potential confounders in the association between social risk and severe vision impairment.

**Table 1:** Indicators and response criteria for five social risk index domains.

SDoH category	Criteria for classification
Economic instability: financial or food insecurity	“Yes” to: “In the past 12 months, have you lost employment or had hours reduced?”
	“Always” or “Usually” to: “During the past 12 months, how often did the food that you bought not last, and you did not have money to get more?”
Low educational attainment	“Never attended school” or “Never graduated high school” to: “What is the highest grade of school you completed?”
Limited health care access	“Yes” to: “Was there a time in the past 12 months when you needed to see a doctor but could not because you could not afford it?”
Unstable built environment: housing and/or transportation insecurity	“Yes” to: “During the last 12 months, was there a time when you were not able to pay your mortgage, rent, or utility bills?”
	“Yes” to: “During the past 12 months, has a lack of reliable transportation kept you from medical appointments, meetings, work, or from getting things needed for daily living?”
Insufficient social support	“Rarely” or “Never” to: “How often do you get the social and emotional support you need?”

SDoH, social determinants of health.

### 2.3 Statistical analysis

Each SDoH domain was coded as a binary variable (present/absent). For categories with multiple items, responding yes to at least one question was defined as having met the criteria for that category. The SDoH measures were then summed across the 5 SDoH domains to construct a Social Risk Index, from 0 (no social risk factor) to ≥4 (four or five social risk factors). Descriptive statistics are presented as unweighted counts. All percentages are survey weighted. Regression analyses applied BRFSS sampling weights to account for the complex survey design (14).

Modified Poisson regression models with robust variance estimation were used to assess associations of each SDoH category and the Social Risk Index, with self-reported severe vision impairment. All models were adjusted for potential confounders selected *a priori* based on prior literature, including age, sex, marital status, and insurance coverage, and incorporated the final BRFSS analytic weights (4). In a sensitivity analysis, we examined whether the associations of interest varied between adults <65 years old and adults ≥65 years old. Additionally, we evaluated whether distinct profiles of social risk were associated with self-reported severe vision impairment. We conducted a latent class analysis using the five binary SDoH domains as class indicators. Models with two to five latent classes were estimated. Each model was fit with 50 random starts, a maximum of 1,000 iterations and evaluated based on Akaike information criterion, Bayesian information criterion, class interpretability, and adequate class sizes. A 3-class model was selected. Classes were labeled based on item-response probabilities for each SDoH domain as: low social risk, medium social risk (economic and built-environment hardship), and high multi-domain social risk. Respondents were assigned to the class with the highest posterior probability. Associations between latent class membership and self-reported severe vision impairment were then estimated using survey-weighted modified Poisson regression adjusted for age, sex, marital status, and insurance coverage, with the lowest-risk class used as the reference group. No adjustment for multiplicity was performed for this exploratory, hypothesis-generating study.

Missing responses were excluded only from analyses requiring the corresponding variable; respondents with missing income were retained in primary models because income was not included as a covariate. Adjusted prevalence ratios (aPR) and corresponding confidence intervals (CI) were reported. Analyses were performed using Stata for data preparation and R 4.5.1 for modeling. Ethical approval was not required for this secondary analysis of publicly available, de-identified BRFSS data.

## 3 Results

Of the 4,558 respondents, 880 (19.8%) reported severe vision impairment in the 2023 Puerto Rico BRFSS. Among all respondents, 2,848 (53.3%) were female and the mean age was 50.2 years (standard error, 0.4), with 1,233 (26.9%) aged 65 years or older. Compared with respondents without severe vision impairment, those with severe vision impairment were more likely to be aged 65 years or older (34.9% vs. 24.9%), female (58.7% vs. 51.9%), have household income less than $15,000 (36.7% vs. 23.3%), be unable to work (12.2% vs. 5.1%), be insured through Medicaid/CHIP (45.2% vs. 38.4%) or Medicare/Medigap (29.5% vs. 20.4%), have a personal health care provider (90.9% vs. 86.1%), and report fair or poor general health (55.8% vs. 29.1%) (Table 2).

**Table 2:** Demographic characteristics of individuals from Puerto Rico in the 2023 BRFSS sample.

Characteristic	Self-reported severe vision impairment						*p-*value
	Overall (*n* = 4,558)		No (*n* = 3,678)		Yes (*n* = 880)		
	*n*	Weighted %	*n*	Weighted %	*n*	Weighted %	
Age group (y)							<0.001
18–24	275	11.2%	243	12.5%	32	5.8%	
25–34	632	15.4%	560	16.6%	72	10.6%	
35–44	659	14.4%	567	15.2%	92	11.1%	
45–54	842	15.7%	665	15.0%	177	18.5%	
55–64	917	16.4%	716	15.7%	201	19.1%	
≥65	1,233	26.9%	927	24.9%	306	34.9%	
Sex							0.01
Male	1,710	46.7%	1,426	48.1%	284	41.3%	
Female	2,848	53.3%	2,252	51.9%	596	58.7%	
Household income ($)							<0.001
<15,000	1,024	26.0%	747	23.3%	277	36.7%	
15,000–24,999	907	19.2%	716	19.2%	191	19.1%	
25,000–34,999	606	11.8%	505	12.3%	101	9.8%	
35,000–49,999	540	11.0%	457	11.6%	83	8.5%	
≥50,000	598	11.2%	542	12.5%	56	5.7%	
Missing	883	20.9%	711	21.0%	172	20.2%	
Employment status							<0.001
Employed	2,181	47.5%	1,884	50.6%	297	34.8%	
Unemployed	292	6.1%	231	6.0%	61	6.7%	
Unable to work	287	6.5%	184	5.1%	103	12.2%	
Other	1,785	39.7%	1,366	37.9%	419	46.3%	
Missing	13	0.4%	13	0.5%	0	0.0%	
Health insurance							<0.001
Employer plan	667	13.7%	598	15.2%	69	7.5%	
Private plan	880	17.8%	762	19.4%	118	11.3%	
Medicare/Medigap	1,023	22.2%	773	20.4%	250	29.5%	
Medicaid/CHIP	1,736	39.7%	1,342	38.4%	394	45.2%	
No coverage	177	4.4%	142	4.3%	35	5.1%	
Other	52	1.4%	40	1.5%	12	1.3%	
Missing	23	0.8%	21	0.9%	2	0.2%	
Has a personal health care provider							0.013
Yes	4,016	87.1%	3,203	86.1%	813	90.9%	
No	528	12.6%	465	13.6%	63	8.6%	
Missing	14	0.3%	10	0.2%	4	0.4%	
General health status							<0.001
Excellent/very good	1,452	31.7%	1,312	35.5%	140	16.5%	
Good	1,599	33.9%	1,354	35.4%	245	27.7%	
Fair/poor	1,507	34.4%	1,012	29.1%	495	55.8%	

Values are unweighted counts, and percentages are survey-weighted. Weighted frequencies incorporated the BRFSS analytic weights to represent the broader Puerto Rico population. Missing values were excluded from statistical tests. Household income reflects respondents’ self-reported annual household income from all sources. For health insurance, “Other” includes military-related coverage (TRICARE/CHAMPUS/VA) and other government programs not listed separately. For employment status, “Other” includes retired, student, or homemaker. *p-*values compare the two visual impairment strata and were calculated using Pearson χ^2^ test with Rao–Scott adjustment; significance threshold α = 0.05.

Across all domains, respondents who reported an SDoH challenge consistently showed a greater share of severe vision impairment compared with those who did not report that challenge. Differences were observed for housing and/or transportation insecurity (33.6% vs. 16.1%), limited health care access (34.1% vs. 18.6%), economic instability (29.0% vs. 17.5%), insufficient social support (27.4% vs. 18.4%), and low educational attainment (29.9% vs. 17.5%). When controlling for age, sex, marital status, and insurance coverage, each individual social risk factor category was significantly associated with self-reported vision impairment. Associations of greater magnitude were observed for housing and/or transportation insecurity (aPR, 2.02; 95% CI, 1.70–2.40), limited health care access (aPR, 1.95; 95% CI, 1.53–2.50), and economic instability (aPR, 1.68; 95% CI, 1.40–2.01), whereas associations of smaller magnitude were observed for insufficient social support (aPR, 1.33; 95% CI, 1.10–1.61) and low educational attainment (aPR, 1.30; 95% CI, 1.06–1.60) (Table 3). Age-stratified analyses were similar (Supplementary Table S1).

**Table 3:** Association between SDoH and self-reported severe vision impairment in Puerto Rico, 2023.

SDoH category	Fraction with vision impairment		aPR (95% CI)^a^
	SDoH present	SDoH absent	
Unstable built environment: housing and/or transportation insecurity	306/979 (33.6%)	560/3,503 (16.1%)	2.02 (1.70–2.40)
Limited health care access	111/334 (34.1%)	769/4,222 (18.6%)	1.95 (1.53–2.50)
Economic instability	246/908 (29.0%)	622/3,583 (17.5%)	1.68 (1.40–2.01)
Insufficient social support	201/724 (27.4%)	667/3,767 (18.4%)	1.33 (1.10–1.61)
Low educational attainment	148/511 (29.9%)	731/4,035 (17.5%)	1.30 (1.06–1.60)

CI, confidence interval; aPR, adjusted prevalence ratio; SDoH, social determinants of health. Percentages are weighted and may not correspond to values obtained by dividing raw counts. ^a^Estimated from separate survey-weighted modified Poisson regression models, adjusted for age, sex, marital status, and insurance coverage.

Among the 4,558 respondents, 2,391 (46.9%) had no social risk factors according to the classification used in this study, whereas 1,278 (31.0%) had a single social risk factor, 577 (13.7%) had two factors, 233 (6.0%) had three factors, and 79 (2.4%) had ≥4 factors. The fraction of respondents with severe vision impairment increased monotonically across levels of the Social Risk Index: 338 of 2,391 (12.9%) respondents with a score of zero reported severe vision impairment, compared with 37 of 79 (45.2%) respondents with a score of four or greater. Compared with those with no risk factors, respondents with one social risk had an aPR of 1.28 (95% CI, 1.03–1.59) and those with ≥4 social risks had an aPR of 3.00 (95% CI, 2.13–4.23) (Table 4). When each of the 5 SDoH were modeled in a latent class analysis, a 3-class model was most parsimonious, representing low-, medium-, and high-risk groups (Supplementary Table S2). Latent class regression found an increased risk of self-reported severe vision impairment in the medium-risk (aPR, 1.96; 95% CI, 1.49–2.56) and high-risk (aPR, 2.23; 95% CI, 1.83–2.72) groups compared with the low-risk group.

**Table 4:** Association between cumulative SDoH and self-reported severe vision impairment in Puerto Rico, 2023

Social risk index	Impairment within this SRI level (Fraction with vision impairment)	aPR (95% CI) ^a^
0	338/2,391 (12.9%)	Reference
1	239/1,278 (19.3%)	1.28 (1.03–1.59)
2	176/577 (31.0%)	2.08 (1.65–2.62)
3	90/233 (40.9%)	2.67 (2.05–3.49)
≥4	37/79 (45.2%)	3.00 (2.13–4.23)

CI, confidence interval; aPR, adjusted prevalence ratio; SDoH, social determinants of health; SRI, Social Risk Index. Fractions represent the number of respondents reporting severe vision impairment divided by the total respondents in that SRI category. Percentages are weighted and may not correspond to values obtained by dividing raw counts. ^a^Estimated from survey-weighted modified Poisson regression models adjusted for age, sex, marital status, and insurance coverage.

## 4 Discussion

This study of 2023 BRFSS data from Puerto Rico revealed that the presence of risk factors in each of five pre-specified SDoH domains (economic instability, low educational attainment, limited health care access, unstable built environment, and insufficient social support) was associated with a higher prevalence of severe vision impairment. As the number of social risk factors increased, the prevalence of self-reported severe vision impairment increased in a graded fashion.

Our results are directionally consistent with previous studies using similar methodologies, although differences in vision impairment prevalence and in the measure of association used limit direct comparison of effect magnitudes. For example, Sharpe et al. compared pilot data from the SDoH module collected across 17 U. S. states in 2017 (4). The authors developed a social risk index incorporating food insecurity, financial strain, neighborhood safety, housing insecurity, and limited health care access. Many of the questions used to construct this index were later modified, replaced, or removed when the BRFSS re-released the SDoH module in 2022, limiting direct comparability across studies. The SDoH categories used in our analysis follow the standardized Healthy People 2030 framework, reflecting a current and nationally aligned approach to measuring social determinants (2). The only domain measured equivalently across both datasets was limited health care access, which was assessed using an unchanged survey item. Sharpe et al. reported that limited health care access was associated with higher odds of vision disability [odds ratio (OR), 1.61; 95% CI, 1.37–1.89], and a cumulative social risk index showed a graded relationship with vision disability (ORs 1.25, 1.57, 1.95, and 2.61 for one, two, three, and ≥4 risks, respectively). These estimates follow the same direction as those observed in our study. Despite methodological differences, both studies show a consistent graded association between cumulative social disadvantage and severe vision impairment.

Besagar et al. reported consistent findings for individual SDoH domains in a recent national analysis of the core BRFSS questionnaire from 2019 to 2020 (5). Using the same BRFSS outcome measure and health care access hardship item, they found that adults unable to afford medical care had higher odds of severe vision impairment (OR, 1.62; 95% CI, 1.51–1.73). Their study likewise showed elevated odds among adults who did not graduate high school (OR, 1.50; 95% CI, 1.38–1.64), a directionally similar association to that observed in our study for low educational attainment (aPR, 1.30; 95% CI, 1.06–1.60). Interpretation across studies should consider that severe vision impairment was relatively uncommon in both Sharpe and Besagar (~6%), making odds ratios a reasonable approximation of relative risk. By contrast, the higher survey-weighted prevalence in our sample (19.8%) makes prevalence ratios the more appropriate and interpretable measure of association.

Findings from Puerto Rico may inform U. S. public health practice more broadly. Puerto Rico represents a setting in which social risk factors cluster at relatively high levels compared with many mainland states. Examining how SDoH relate to vision impairment in this context may help U. S. health systems better understand how these determinants operate in other culturally, linguistically diverse, or economically disadvantaged communities across the country.

It is important to emphasize that no inferences about causality can be made from this cross-sectional analysis. While it is possible that one or more SDoH precede the onset of severe vision impairment, it is also possible that individuals with visual disabilities are more likely to encounter adverse SDoH as a result of their condition. Thus, SDoH may be both a cause and a consequence of severe vision impairment (19–21). SDoH are modifiable socioeconomic environments and could be targets for public policy, but it is not clear that eliminating one or more of these social risk factors would improve vision. Nonetheless, identification of social risk factors is important since eye care interventions could be directed toward populations carrying a high burden of social risk. Moreover, the results of this study suggest that it may be important to consider the cumulative burden of multiple SDoH rather than considering them as individual forces.

Previous work has suggested it may be possible to reduce preventable vision loss and improve eye health by targeting social and structural barriers. Williams and Sahel have encouraged ophthalmologists to address SDoH through a five-part framework: awareness, assistance, adjustment, alignment, and advocacy (22). This framework is guided by recommendations from the National Academies of Sciences, Engineering, and Medicine and has already been successfully applied across various medical specialties (23). They suggest actionable strategies such as using standardized social screening forms that integrate with electronic health records, hiring patient navigators to connect individuals with community resources, offering evening and telemedicine appointments for patients with scheduling or transportation barriers, and partnering with local organizations to provide mobile vision screenings. Others have established frameworks to help ophthalmologists address the SDoH through community engagement and multisector partnerships (24).

This study has limitations. As stated previously, the cross-sectional observational study design of BRFSS limited the ability to establish causal relationships between social risk factors and disability. A limited set of potential covariates was available in the BRFSS, so residual confounding is possible. The reliance on self-reported data may have resulted in misclassification of both exposures and outcomes, which could have introduced recall and response biases. The direction of any misclassification is not known. If respondents with greater social risk were also more likely to report poor health in general, the associations would be overstated. If misreporting occurred equally across social risk groups, the associations would instead be understated.

The outcome was measured via a single self-reported item collected without examination-based confirmation. It is noteworthy that Alfaro Hudak et al. found SDoH to have similar associations with both self-reported and clinical visual impairment, providing at least some support for using the self-reported outcome in this study (3). Examination-based population estimates of visual impairment are not available for Puerto Rico, so surveillance in this setting is only possible using self-reported measures. While prior work has suggested that self-reported vision questions may not be sufficiently accurate to serve as a diagnostic test at the individual level (e.g., AUC 0.80), responses to the particular blindness question used by the BRFSS have been shown to be closely correlated with examination-based visual acuity loss at the population-level (18). Prevalence estimated from this item is nonetheless not directly equivalent to prevalence based on best-corrected visual acuity, and the item cannot separate uncorrected refractive error and other correctable causes from irreversible vision loss. Estimates reported here should therefore be interpreted as the prevalence of self-reported severe vision impairment rather than of examination-based visual impairment.

The BRFSS samples only noninstitutionalized adults and therefore excludes residents of nursing homes and long-term care facilities, among whom both vision impairment and social risk are likely to be more common. Therefore, it is possible that the prevalence reported here is an underestimate of the prevalence of severe vision impairment among all adults in Puerto Rico. Finally, the findings of this study may not be generalizable to populations beyond adults residing in Puerto Rico, limiting their applicability to other demographic or geographic contexts.

In summary, self-reported severe vision impairment among adults was high in the 2023 BRFSS data from Puerto Rico, and associated with several SDoH. These analyses support using SDoH indicators to direct eye care interventions to the populations in highest need.

## Data Availability

The original contributions presented in the study are included in the article/Supplementary material, further inquiries can be directed to the corresponding author.
