# Supplementary material for: Severe vision impairment is associated with cumulative social risk among adults in Puerto Rico: a population-based study
Source: Front Public Health. 2026 Sep 16;14:1907768. doi: 10.3389/fpubh.2026.1907768 (PMC13623729; doi:10.3389/fpubh.2026.1907768)
Supplement: Supplementary file 1 [file Data_sheet_1.pdf]

Table S1. Association between SDoH and self-reported severe vision impairment stratified by age group (<65 versus ≥65 year of age) in Puerto Rico, 2023.

SDoH category	Age group	Fraction with vision impairment		aPR (95% CI)[a]
		SDoH present	SDoH absent	
Unstable built environment: housing and/or transportation insecurity	<65	234/817 (30.1%)	331/2444 (13.8%)	1.93 (1.57–2.38)
	≥65	72/162 (49.5%)	229/1059 (21.5%)	2.28 (1.70–3.05)
Limited health care access	<65	94/300 (31.4%)	480/3023 (16.2%)	1.87 (1.41–2.49)
	≥65	17/34 (59.0%)	289/1199 (24.7%)	2.25 (1.52–3.34)
Economic instability	<65	204/798 (27.3%)	362/2471 (14.7%)	1.65 (1.35–2.03)
	≥65	42/110 (39.5%)	260/1112 (23.9%)	1.66 (1.14–2.41)
Insufficient social support	<65	139/516 (25.4%)	427/2753 (16.4%)	1.40 (1.11–1.77)
	≥65	62/208 (31.7%)	240/1014 (24.1%)	1.27 (0.91–1.77)
Low educational attainment	<65	61/214 (29.7%)	513/3103 (16.4%)	1.42 (1.06–1.89)
	≥65	87/297 (30.0%)	218/932 (22.3%)	1.30 (0.97–1.75)

Abbreviations: CI = confidence interval; aPR = adjusted prevalence ratio; SDoH = social determinants of health
Percentages are weighted and may not correspond to values obtained by dividing raw counts.
[a] Estimated from survey-weighted modified Poisson regression models, adjusted for age, sex, marital status, and insurance coverage.

Table S2. Association between social risk profiles and self-reported severe vision impairment in Puerto Rico, 2023.

Characteristic	Vision impairment within class	aPR (95% CI)	p-value
Latent social risk class			
Low social risk	16.7%	Reference	
Economic and built-environment hardship	30.0%	1.96 (1.49–2.56)	<0.001
High multidomain social risk	40.1%	2.23 (1.83–2.72)	<0.001

Abbreviations: CI = Confidence Interval, aPR = Adjusted Prevalence Ratio.
